# A Successful En Bloc Excision of a Giant Retroperitoneal Liposarcoma With Distal Splenopancreatectomy

**DOI:** 10.7759/cureus.18903

**Published:** 2021-10-19

**Authors:** Leena H Moshref

**Affiliations:** 1 Surgery, Doctor Soliman Fakeeh Hospital, Jeddah, SAU

**Keywords:** excision, magnetic resonance imaging, computed tomography, en bloc, retroperitoneal liposarcoma

## Abstract

Retroperitoneal sarcomas are considered a rare type of malignant tumors, accounting for fewer than one-fifth of all cases. Among all malignancies, the incidence of soft tissue sarcomas is approximately 1%. It constitutes 38% of soft tissue sarcomas in liposarcomas. Putting it that way may better indicate that liposarcomas are a rare tumor. They are frequently asymptomatic until they become large enough to compress the adjacent organs. As a result, it must be validated using appropriate imaging modalities, for example, computed tomography (CT) abdomen or magnetic resonance imaging (MRI) abdomen with contrast. The preferred treatment is complete surgical resection.

The patient presented with vague symptoms. She had a CT abdomen with contrast, as well as an MRI abdomen. Both images revealed the presence of a giant sarcoma displacing the pancreas and left kidney posteriorly and the transverse colon inferomedially. The patient underwent en bloc resection of the mass with distal splenopancreatectomy. The patient tolerated the procedure well and was discharged on day 6 postoperatively in a stable state.

Giant liposarcoma is a rare and aggressive form of sarcoma. Because of the unique presentation, it is difficult to diagnose clinically. CT scans with MRIs are viable imaging modalities for determining tumor extent and ruling out any vascular invasion. Complete surgical resection of liposarcoma is a treatment of choice. En bloc resection of retroperitoneal sarcoma with distal splenopancreatectomy can be performed successfully and safely.

## Introduction

Retroperitoneal sarcomas are considered a rare type of malignant tumors, accounting for less than one-fifth of cases. Liposarcoma is the most prevalent kind of sarcoma, accounting for 40% of all cases [1].

They are frequently asymptomatic until they become large enough to compress the adjacent organs, thus producing symptoms [2]. Therefore, it needs to be confirmed by appropriate imaging modalities, for example, computed tomography (CT) abdomen or magnetic resonance imaging (MRI) abdomen with contrast [3].

Retroperitoneal liposarcoma involving the spleen and tail of the pancreas has been reported in few studies [4-7]. Furthermore, only 14 cases of giant retroperitoneal liposarcoma have been reported in the literature (diameter greater than 30 cm) [8].

This is a case of a giant retroperitoneal liposarcoma including the spleen and tail of the pancreas that was effectively treated with en bloc resection.

## Case presentation

Case history and examination

A 36-year-old female presented to the clinic with a six-month history of abdominal distension. Examination revealed abdominal distension and ill-defined mass.

**Figure 1:**
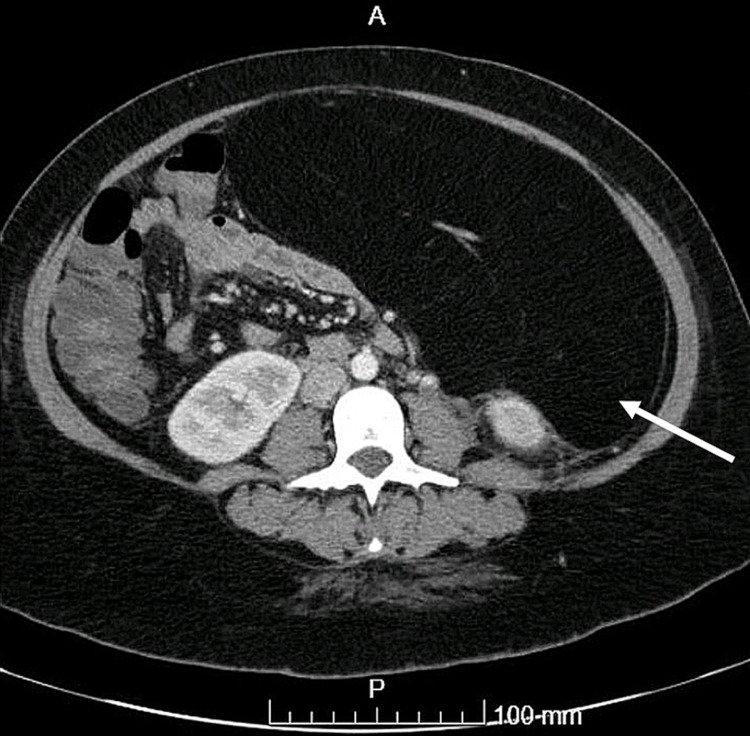
CT chest, abdomen, and pelvis for staging: large fat-containing lesion (arrow) seen intra-abdominally extending from the left upper quadrant.

**Figure 2:**
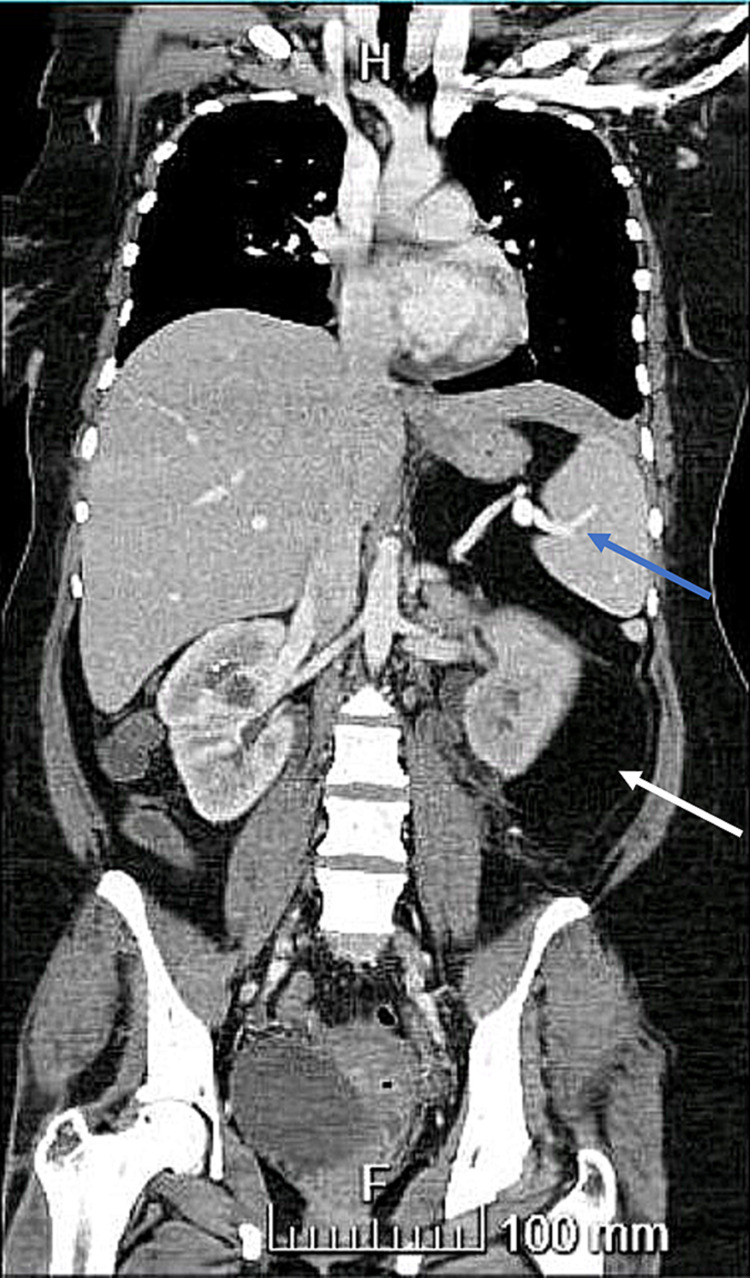
CT chest, abdomen, and pelvis for staging: large fat-containing lesion (white arrow) seen intra-abdominally extending from the left upper quadrant. The splenic artery and vein run within the lesion centrally to the splenic hilum (blue arrow) with no compression, infiltration, thrombosis, or occlusion.

A CT chest, abdomen, pelvis for staging was done (Figures 1, 2): fat-containing lesion seen intra-abdominally extending from the left upper quadrant likely extending 30 cm in a craniocaudal (CC) direction and measuring 10 cm*22 cm in the antero-posterior (AP) and transverse diameters. The lesion is well encapsulated and is causing significant displacement of the small and large bowel loops posteriorly and to the right-side abdomen. The pancreas and the left kidney were displaced posteriorly. There is no infiltration into the adjacent structures with no vascular thrombosis or occlusion. The splenic artery and vein run within the lesion centrally to the splenic hilum with no compression, infiltration, thrombosis, or occlusion.

**Figure 3:**
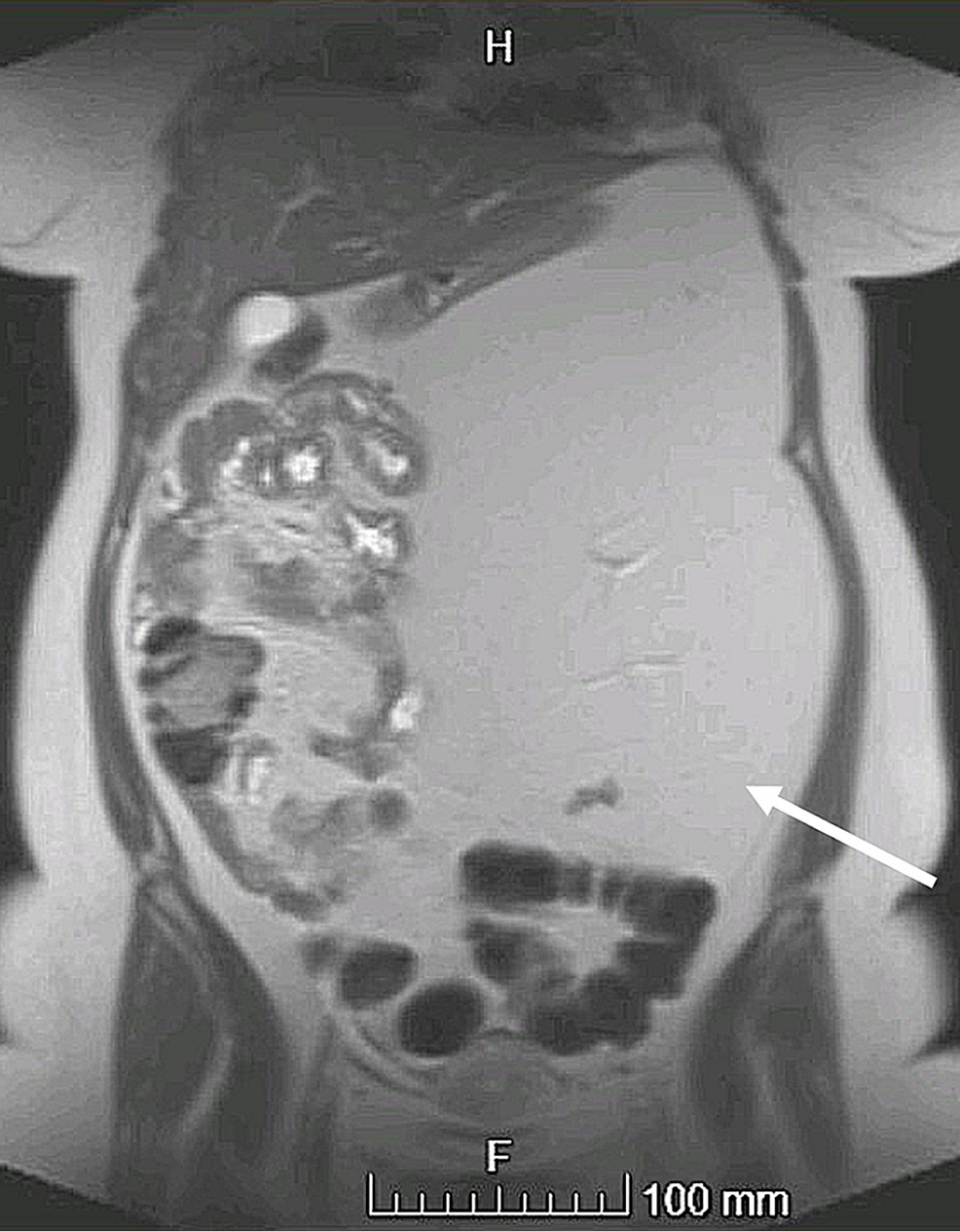
MRI abdomen and pelvis with contrast showing a large abdominal mass (arrow) in the left side of the abdominal cavity with homogeneously high in T1 and T2 WI and homogenous drop of the signal in the fat-sat sequences.

Thus, an MRI abdomen and pelvis with contrast (Figure 3) was done and showed a large abdominal mass in the left side of the abdominal cavity measures around 11.3 x 20 x 30 cm in maximum AP, transverse, and CC diameters respectively, showing homogeneously high in T1 and T2 WI with a homogenous drop of the signal in the fat-sat sequences with the displacement of organs as mentioned in CT.

The case was discussed on the tumor board and agreed that the tumor was most likely G1 liposarcoma that needed oncological excision of retroperitoneal liposarcoma with distal splenopancreatectomy. The patient received presplenectomy immunization (S. pneumoniae, N. meningitidis, H. influenzae).

Operation and postoperative period

The patient was admitted for surgery. Preoperative lab tests (along with procalcitonin and serology) were normal. Antibiotic prophylaxis was given and an epidural catheter was inserted.

Operation

She underwent en bloc resection of the retroperitoneal tumor with distal splenopancreatectomy. Medline laparotomy then exploration showed a retroperitoneal tumor that occupied 70% of the abdominal cavity with transposition of the viscera to the right side, beginning at the lower border of the tumor and dissection from the transverse colon, entry to the bursa omentalis, and identification of the inferior mesenteric vessels retroperitoneally, then further dissection from the caudal to cephalic identifying the left ovarian vein and ureter and both structures followed until the kidney hilum. The mass capsule was surrounding the hilum without invasion. Then, medial dissection up to the inferior pancreatic border was done, which was engulfed by the tumor and could not be easily separated. Then, lateral mobilization of the tumor en bloc with the spleen. This was followed by transection of the gastric greater curvature vessels and identification of the splenic artery and vein on the superior border of the pancreas, which was also engulfed by the mass and disappear in the mass completely.

**Figure 4:**
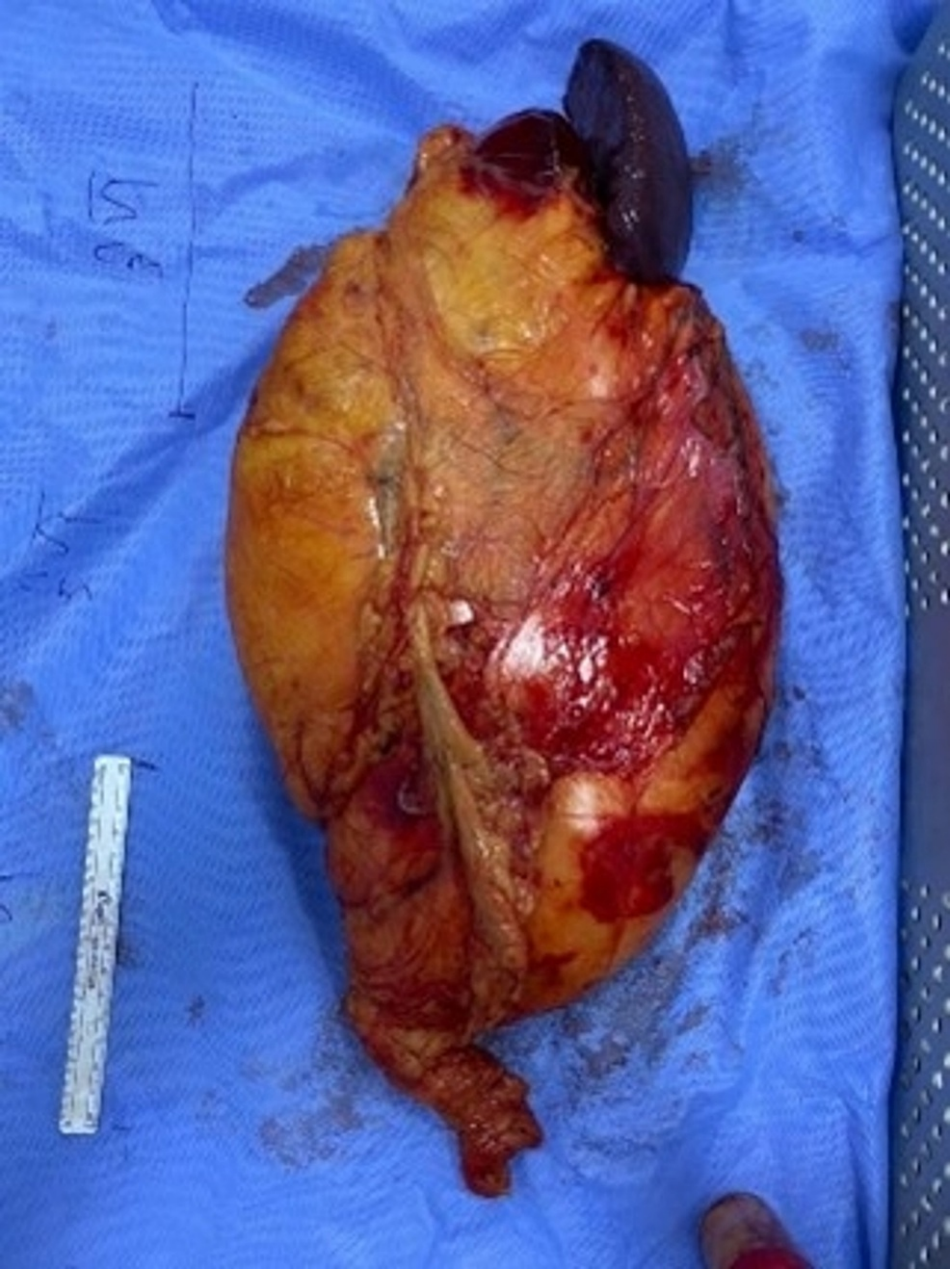
Gross pathology of the specimen showing a large mass with distal pancreas and spleen resected.

After transection of the pancreas tail, the splenic artery and vein were resected. After complete medial mobilization, lateral mobilization was completed by dissection of the mass from the left kidney, adrenal, and diaphragm. The mass was retrieved with an intact capsule and sent for histopathological examination (Figure 4). Two J-VAC drains (JVD) were inserted. This was followed by the closure of the transverse colon window and fixation of the left colon to the lateral peritoneum. Closure of the abdominal layers was done in a similar manner. She was transferred to the post-anesthesia care unit then to the surgical ward in a stable condition.

She was placed postoperatively on analgesia, anti-emetic and anticoagulant prophylaxis.

She was followed up daily. Blood sugar readings were normal during the hospital stay, and there were no attacks of hypoglycemia. In the postoperative phase, laboratory tests were unremarkable. Procalcitonin, amylase, and lipase levels were normal on days 3 and 5 postoperative.

The patient felt attacks of dull abdominal pain upon eating in the left upper quadrant. As a result, an ultrasound of the abdomen and pelvis was performed and showed a left upper abdomen encysted turbid and multiseptated collection measuring 73 x 52 mm diameters and approximately 129 mL volume. The JVD was kept in place and was started on ondansetron regularly. On the sixth postoperative day, the patient was discharged on proton pump inhibitors, analgesia, and antibiotics. The patient was followed one week in the clinic after discharge and it was unremarkable.

Histopathological examination revealed a neoplasm that consisted of adipose tissue with slightly variable in size fat cells and rare fibrous bands with spindle cells that exhibited no atypia. All margins were involved by neoplasm. There was no pathological alteration in the spleen. Mouse double minute 2 homolog (MDM2) was focally weak +ve, and cyclin-dependent kinase 4 (CKD4) was -ve. Diagnosis confirmed a low-grade liposarcoma.

## Discussion

Retroperitoneal sarcomas are rare malignant tumors that account for 15% of all sarcomas. Among all malignancies, the incidence of soft tissue sarcomas is approximately 1%. It constitutes 38% of soft tissue sarcomas in liposarcomas [1]. Retroperitoneal liposarcomas can develop at any age; however, they are more common in the sixth and seventh decades [2]. Our case was in her fourth decade of life. Most of them are malignant except if they were originating from benign lipomas [9]. 

Liposarcomas tend to grow to very large sizes before they produce symptoms due to large retroperitoneal space. When diagnosed, almost half of retroperitoneal sarcomas are larger than 20 cm in size [2]. Most patients are asymptomatic. Nevertheless, if they are symptomatic, they frequently present with unspecific symptoms, for example, flank pain, early satiety, or pain [2]. Similarly, our patient had been experiencing dull abdominal pain for 6 months. Other differential diagnoses must be ruled out, for instance, adrenal/renal/pancreatic cancers, advanced gastrointestinal malignancies, teratomas, and lipoma [9,10].

The most often utilized imaging modality for sarcoma diagnosis, staging, and preoperative evaluation is CT [11,12]. Imaging the abdomen and pelvis aids in determining the location of the sarcoma, and excluding intraperitoneal seeding, regional lymph node involvement, and liver metastasis. A CT scan of the chest is required to rule out the presence of metastases. There have been few studies that suggest the efficacy of MRI in diagnosing retroperitoneal liposarcoma and determining neurovascular and muscle invasion [10]. In our study, a CT staging of the chest, abdominal, pelvis was ordered to determine the extent of the mass and rule out any metastases. It had been demonstrated to be a practical method to see the size of the tumor and if there is any vascular or nearby invasion.

Several prognostic variables for survival and distant and local recurrence have been identified. Complete surgical resection with negative margins is the most powerful prognostic factor [13]. Several studies have demonstrated that the inability to obtain negative margins due to anatomic constraint or local tumor invasion is a negative prognostic factor for disease-specific survival [13]. In our patient, a negative margin was achieved by a splenopancreatectomy as illustrated in imaging that the tumor displaced the pancreas and splenic artery and vein run within the lesion. In addition, intraoperatively, the inferior border of the pancreas could not be separated from the mass, along with its blood supply (splenic artery). Thus, resection of the pancreatic tail and spleen was performed.

Furthermore, histologic grade, which reflects the degree of differentiation, continues to be a crucial component in clinical course and prognosis [14]. The five-year survival rates are over 90% in well-differentiated and decline as the grade climbs [14]. Pathology and immunohistochemical analyses confirm the diagnosis of retroperitoneal liposarcoma [14]. Our patient had a G1 liposarcoma and was completely excised.

## Conclusions

Giant liposarcoma is a rare and aggressive form of liposarcoma. It is hard to diagnose clinically due to atypical presentation. CT scans with MRIs are viable imaging modalities for determining tumor extent and ruling out vascular invasion. The treatment of choice is complete surgical en bloc resection of retroperitoneal sarcoma with distal splenopancreatectomy, which can be performed successfully without complications. However, to detect any vascular invasion, a rigorous preoperative assessment is required.
